# Supplementary material for: Characterization of nucleolar SUMO isopeptidases unveils a general p53-independent checkpoint of impaired ribosome biogenesis
Source: Nat Commun. 2023 Dec 8;14:8121. doi: 10.1038/s41467-023-43751-9 (PMC10709353; doi:10.1038/s41467-023-43751-9)
Supplement: Supplementary file 1 — Supplementary Figures and Legends [file 41467_2023_43751_MOESM1_ESM.pdf]

Supplementary Figure 1

(a) HEK293T cells were transfected for 48 h with FLAG-tagged wild-type SENP3, SENP3^{C532S}, wild-type SENP5, SENP5^{C713S} or empty vector control. After cell lysis immunoblotting against SENP3 or SENP5 was performed. (b) Transfection of HEK293T cells for 48 h with FLAG-tagged SENP3^{C532S} or SENP5^{C713S}. Cells were fixed and analysed by immunofluorescence using FLAG antibody. (c) Whole cell lysates of U2OS WT or U2OS SENP3 KO cells were immunoblotted against SENP3, SUMO2/3 or β-Tubulin. (d) HeLa cells were transfected with two different siRNAs against SENP3, SENP5 or siControl for 72 h. Additionally cells were treated with TAK-981 (200 nM) or DMSO for 4 h directly before cell lysis. Afterwards lysates were immunoblotted against UTP14A or Vinculin. (e) HeLa cells were transfected with siRNAs against SENP3, SENP5, SENP3/5 or siControl. 72 h post transfection, endogenous SUMO2/3 or IgG control IP were performed and samples were analysed by immunoblotting using antibodies against UTP18, BMS1 or SUMO2/3. Input samples are shown in the left panel. SUMOylated proteins are indicated. Source data for a, c, d, e are provided as a Source Data file.

Supplementary Figure 2

(a) Quantification of the UTP14A protein signal in sucrose gradient density centrifugation. For quantification, the signal for UTP14A in each fraction of the gradient was determined and normalized to the input sample (representing 10% of the cleared cell extract that was loaded onto the gradient). After normalisation to the input, a scaling was performed enabling comparison of the different experiments with different overall signal intensities. The blue line represents the data of samples from control cells (siControl), while the red line represents SENP3/5 depleted cells. Two independent replicates were used for quantification. (b) & (c) UTP14A localization upon siRNA mediated depletion of SENP3 or SENP5 in HeLa cells. (b) Representative confocal images. Scale bar = 10 µm. Dashed lines represent the nucleus. (c) Quantification of the nucleolar vs nucleoplasmic ratio of the UTP14A intensity normalized to the mean nuclear intensity. 2-sided t tests were performed. Data are shown as mean ± standard deviation. (d) HEK293T cells were depleted for SENP3 and SENP5 by siRNA transfection for 72h. After 24 h cells were transfected with FLAG-tagged wild-type UTP14A or UTP14^{K733R} encoding plasmids either in combination with an empty vector control or with a His-tagged SUMO2 construct for 48 h. Ni-NTA beads were used to enrich for SUMO2-UTP14A conjugates. Immunoblotting was performed against FLAG-tag, His or β-Tubulin. Input samples are shown in the left panel. The SUMOylated form of UTP14A is indicated by arrows. (e) UTP14A (red) and its main SUMOylation site K733 (yellow) were highlighted in the available SSU processome structure (PDB:7MQ8, https://doi.org/10.2210/pdb7MQ8/pdb). Source data for a, c, d are provided as a Source Data file.

Supplementary Figure 3

(a) Scheme of the pre-rRNA species detected by the 5'ITS1 (blue) or the ITS2 probe (orange), used in Figure 4b. (b) Validation of endogenous Halo-tagging by immunoblotting. Lysates of single clones of RPS3-Halo or RPL28-Halo cells were stained against RPS3 or RPL28. The clones marked with asterisks were chosen for further experiments. (c) Ribo-Halo fusion proteins were modelled into the available 40S and 60S ribosomal subunit structures (PDB:4V6X, https://doi.org/10.2210/pdb4V6X/pdb). (d) Whole-cell lysates from endogenously tagged HeLa cells (RPS3-Halo or RPL28-Halo) were separated by using sucrose density centrifugation. Peaks corresponding to (pre-)ribosomal complexes are marked on an absorbance profile at 260 nm (top). The presence of the indicated proteins in each fraction of the gradient was determined by western blotting using antibodies against Halo, RPL3, RPS9 or RPS3A (bottom). Experiments were performed with two replicates. (e) HeLa RPS3-Halo cells were depleted for SENP3 and SENP5 and were treated with both Halo-ligands.

In subsequent immunofluorescence imaging 'old' 40S ribosomal subunits are shown in red and 'new' 40S ribosomal subunits in green. Representative images are shown (upper panel). Lower panel: Quantification of the intensity of newly synthesized vs old ribosomes in the cytoplasm. 2-sided t testing was performed for statistical analysis. The data are shown as mean ± standard deviation. Source data for b, d, e are provided as a Source Data file.

Supplementary Figure 4

(a) Validation of SENP3 knock-down efficiency of samples used for whole cell proteome analysis (Figure 5a). All replicates used for MS were analysed by immunoblotting against SENP3 and Vinculin. (b), (c) Volcano plots of whole cell proteome analysis in U2OS cells (Figure 5a) transfected with siSENP3-1 (b) or siSENP3-2 (c). Highlighted are hits, that shared the same regulation with both siRNAs (log2 ratio ≥1; -log10 p-value ≥1.3), which were identified based on 2-sided Student´s t test analysis. The cell cycle regulator p21/CDKN1A is additionally highlighted. (d) Example of gating strategy used for PI staining analysis by flow cytometry. Cells were plotted with the FSC-A against the SSC-A channel. The living population was gated and then plotted for the PI-A signal as a histogram. Only PI positive cells were gated. G1, S and G2 phases were determined based on the PI signal. The same gates were transferred to all samples in the same experiment. Source data for a are provided as a Source Data file.

Supplementary Figure 5

(a) Validation of SENP3 knock-down efficiency of samples used for whole cell proteome analysis (Figure 6a). All replicates used for MS were analysed by immunoblotting against SENP3 and Vinculin. (b) & (c) Volcano plots of whole cell proteome analysis in Saos- 2 cells (Figure 6a) transfected with siSENP3-1 (b) or siSENP3-2 (c). Highlighted are hits, that shared the same regulation with both siRNAs (log2 ratio ≥1; -log10 p-value ≥1.3), which were identified based on 2-sided Student´s t test analysis. The cyclin-dependent kinase, CDK6, is additionally highlighted. (d) RNA-Seq analysis of U2OS cells transfected with siSENP3-1 (Y-axis) or siSENP3-2 (X-axis) compared to control siRNA transfection. Down-regulated RNAs are visualized in a XY diagram, comparing either of the two SENP3 siRNAs against the control. Only significant hits (log2 ratio ≥ 1; p-value < 0.05) are displayed. CDK6 is highlighted in red. Experiments were performed with four replicates. (e) U2OS cells were transfected with siControl or two different siRNAs against SENP3 for 72 h. MG132 (10 µM) or DMSO was added for 15 h before lysing the cells. Lysates were immunoblotted against CDK6 or Vinculin. (f) Whole cell lysate samples from Fig. 6f were immunoblotted against SENP5, SENP3, Vinculin or β-Tubulin. Source data for a, e, f are provided as a Source Data file. * unspecific signal.

Supplementary Figure 6

(a) PI staining performed as shown before in Figure 7a in parental RPE1 cells (left) or RPE1$^{\Delta p53}$ cells (right) transfected with siCDK6 (green) or siControl (blue) for 72 h. Significance level was calculated by 2-sided t testing. Experiments were performed with four replicates and are shown as mean ± standard deviation. Mean values of percentages are added above the bars. (b) Proliferation assay of parental RPE1 cells (left) or RPE1$^{\Delta p53}$ cells (right) transfected with siSENP3 or siControl. The cell index was measured by an XCELLigence RTCA SP instrument for 100 h. Experiments were performed as duplicates and the mean value of both replicates is shown here. (c) Cell cycle analysis using PI staining of U2OS cells transfected for 72 h with siRNAs as indicated. Percentages of cells in the S phase were determined and significance level was calculated using 2-sided t testing. Experiments were performed with four replicates and are shown as mean ± standard deviation. (d) Whole cell lysates related to Figure 7c were immunoblotted against phospho-RB (S807/811). (e) U2OS cells stably expressing FLAG-CDK6 under a doxycycline-inducible promoter were transfected with control siRNA or siRNA against SENP3 (siSENP3-2) for 72 h. FLAG-CDK6 expression was induced by supplementing

the media with doxycycline (0.05 µg/ml) 15 h before cell lysis. Immunoblots were performed as indicated. Source data for a-e are provided as a Source Data file.

Supplementary Figure 7

(a) U2OS cells or (b) Saos-2 cells were transfected with the indicated siRNAs. 72 h after transfection cells were lysed and lysates were immunoblotted against CDK6 or Vinculin. (c) Lysates related to Figure 9 b, c were immunoblotted as indicated to validate proper efficiency of protein depletion. (d) Parental RPE1 cells were transfected for 72h with the indicated siRNAs. Cell lysates were analysed by immunoblotting as indicated. Source data for a-d are provided as a Source Data file.

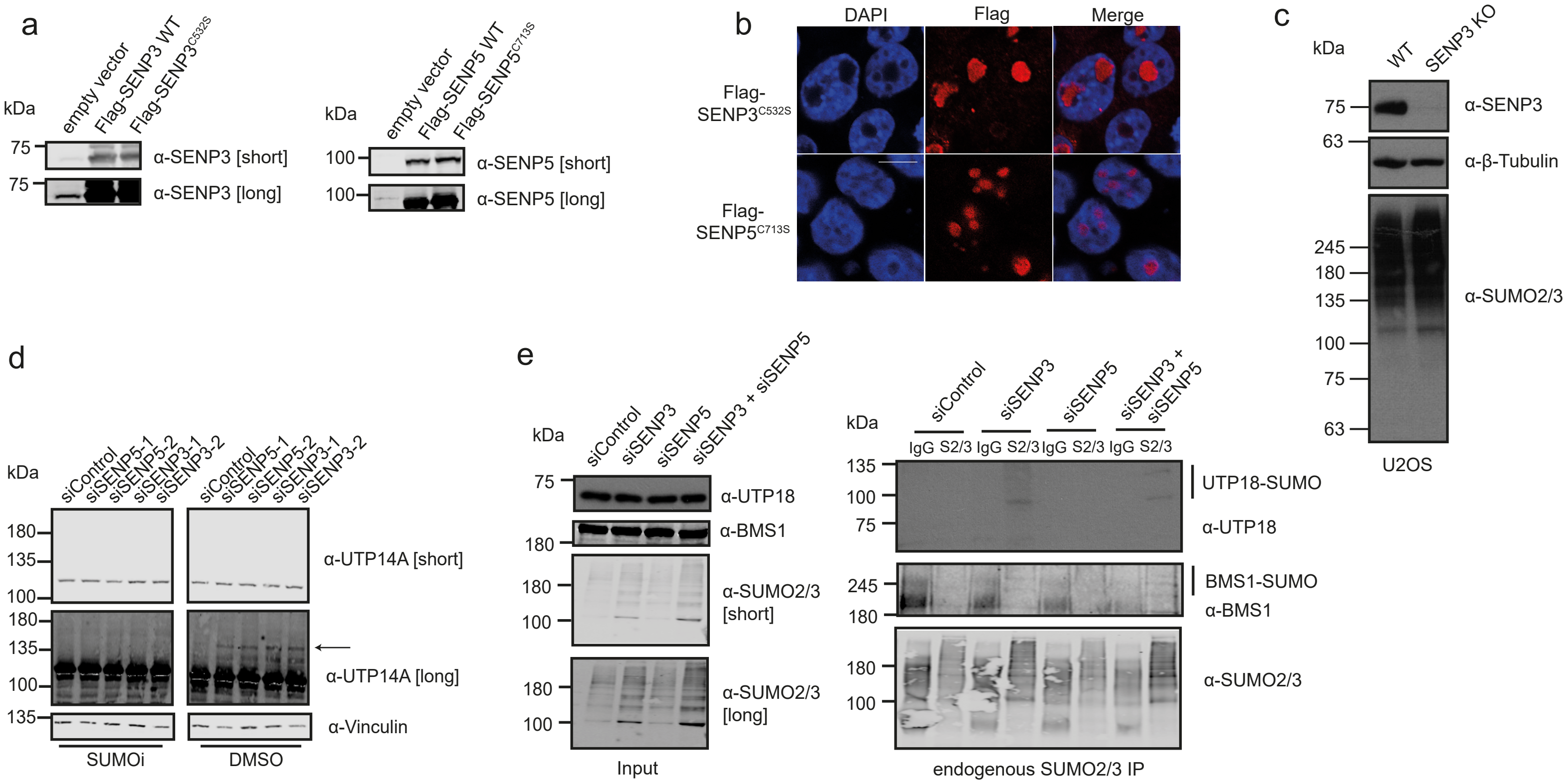

a
kDa
empty vector
Flag-SENP3 WT
Flag-SENP3C532S
75
α-SENP3 [short]
α-SENP3 [long]
empty vector
Flag-SENP5 WT
Flag-SENP5C713S
100
α-SENP5 [short]
α-SENP5 [long]
b
DAPI
Flag
Merge
Flag-SENP3C532S
Flag-SENP5C713S
c
WT
SENP3 KO
α-SENP3
α-β-Tubulin
α-SUMO2/3
245
180
135
100
63
U2OS
d
siControl
siSENP5-1
siSENP5-2
siSENP3-1
siSENP3-2
α-UTP14A [short]
α-UTP14A [long]
α-Vinculin
SUMOi
DMSO
e
siControl
siSENP3
siSENP5
siSENP3 + siSENP5
α-UTP18
α-BMS1
α-SUMO2/3 [short]
α-SUMO2/3 [long]
Input
IgG
S2/3
UTP18-SUMO
BMS1-SUMO
endogenous SUMO2/3 IP

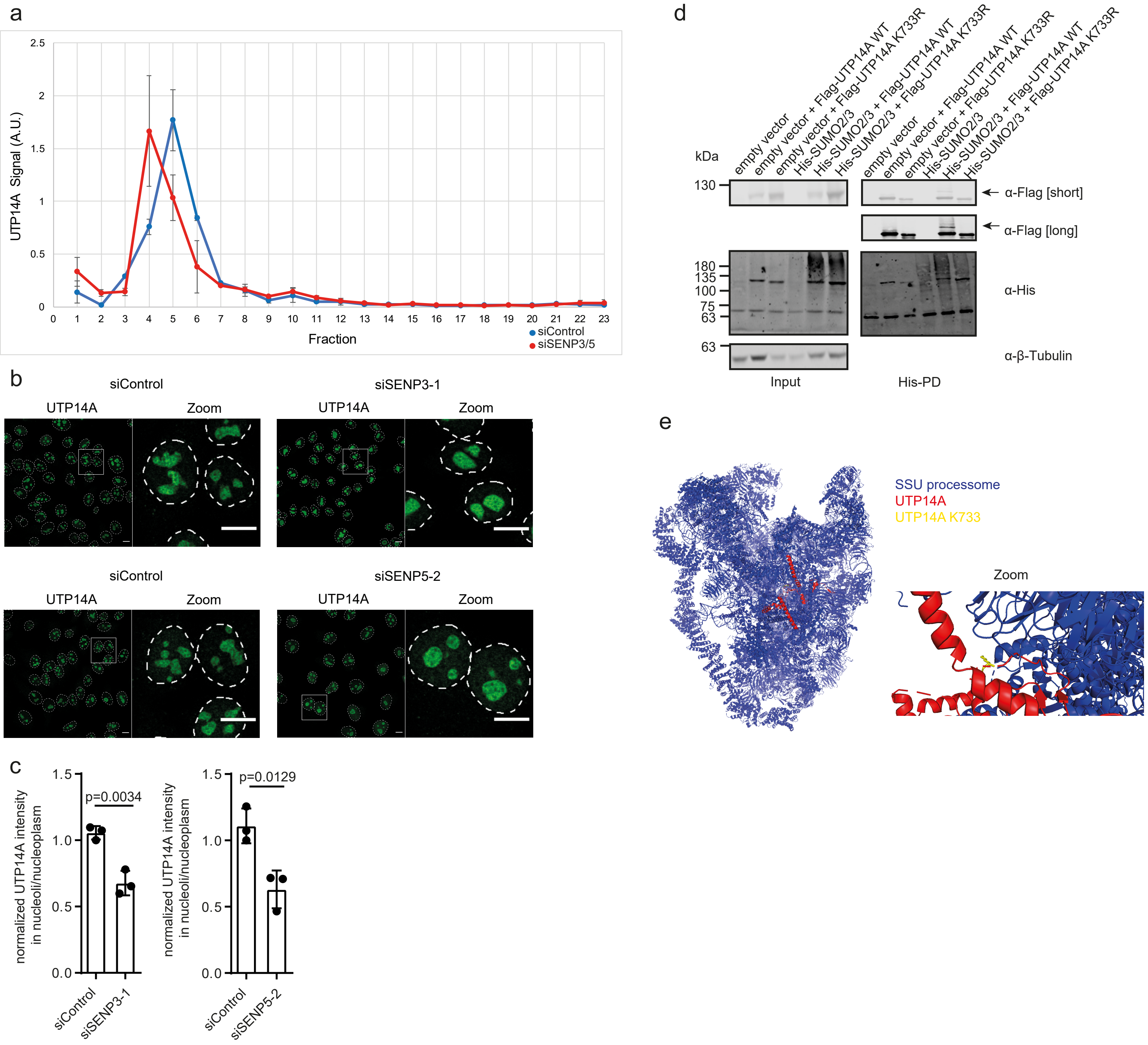

a
UTP14A Signal (A.U.)
2.5
2
1.5
1
0.5
0
0 1 2 3 4 5 6 7 8 9 10 11 12 13 14 15 16 17 18 19 20 21 22 23
Fraction
siControl
siSENP3/5
b
siControl
UTP14A
Zoom
siSENP3-1
UTP14A
Zoom
siControl
UTP14A
Zoom
siSENP5-2
UTP14A
Zoom
c
normalized UTP14A intensity in nucleoli/nucleoplasm
1.5
1.0
0.5
0.0
p=0.0034
siControl
siSENP3-1
normalized UTP14A intensity in nucleoli/nucleoplasm
1.5
1.0
0.5
0.0
p=0.0129
siControl
siSENP5-2
d
empty vector
empty vector + Flag-UTP14A WT
empty vector + Flag-UTP14A K733R
His-SUMO2/3
His-SUMO2/3 + Flag-UTP14A WT
His-SUMO2/3 + Flag-UTP14A K733R
empty vector
empty vector + Flag-UTP14A WT
empty vector + Flag-UTP14A K733R
His-SUMO2/3
His-SUMO2/3 + Flag-UTP14A WT
His-SUMO2/3 + Flag-UTP14A K733R
kDa
130
α-Flag [short]
α-Flag [long]
180
135
100
75
63
α-His
63
α-β-Tubulin
Input
His-PD
e
SSU processome
UTP14A
UTP14A K733
Zoom

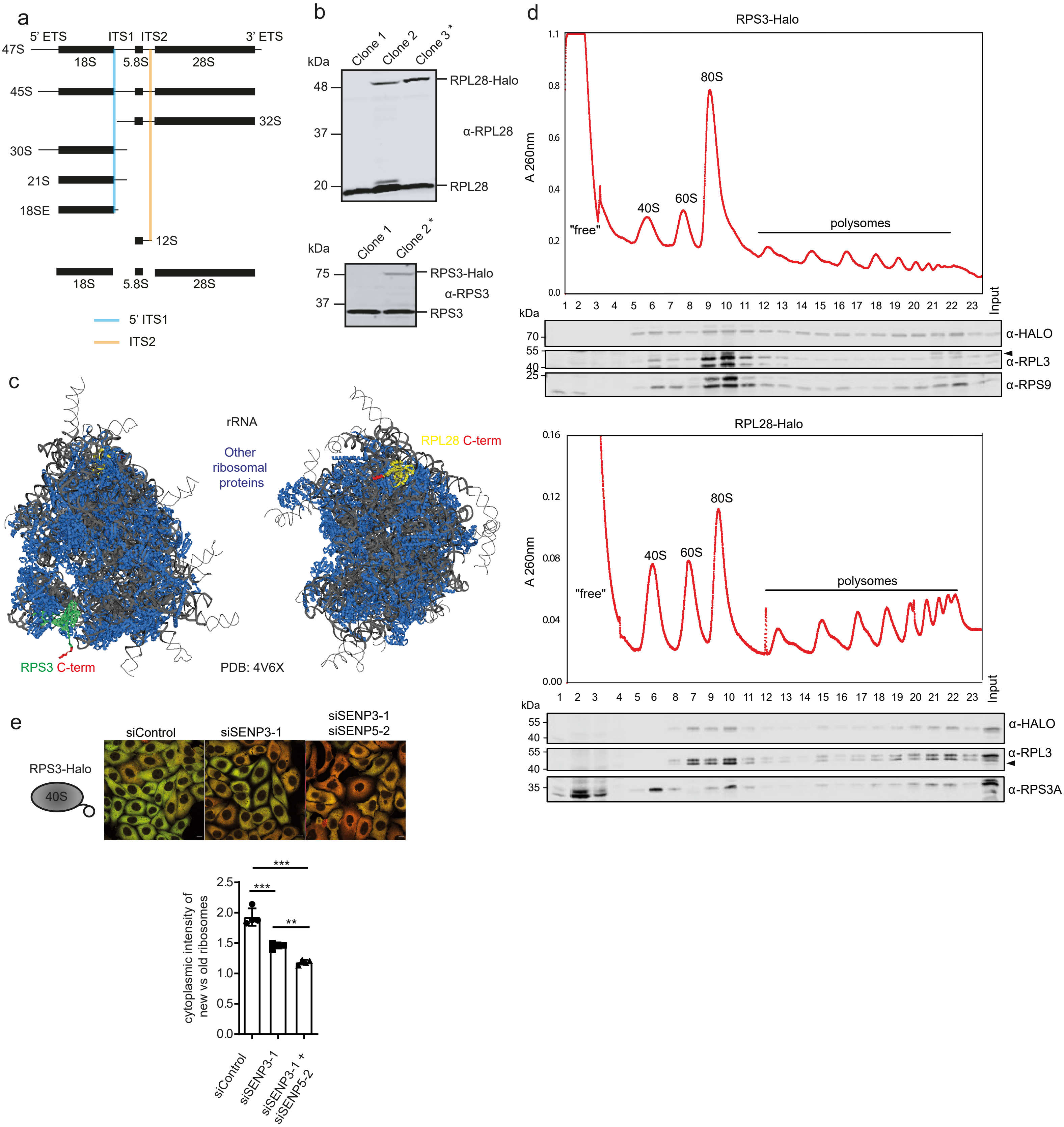
a
5' ETS
ITS1
ITS2
3' ETS
47S
18S
5.8S
28S
45S
32S
30S
21S
18SE
12S
5' ITS1
ITS2
b
kDa
Clone 1
Clone 2
Clone 3 *
48
37
20
RPL28-Halo
α-RPL28
RPL28
Clone 2 *
75
RPS3-Halo
α-RPS3
RPS3
c
rRNA
Other ribosomal proteins
RPL28 C-term
RPS3 C-term
PDB: 4V6X
d
RPS3-Halo
A 260nm
"free"
40S
60S
80S
polysomes
Input
α-HALO
α-RPL3
α-RPS9
RPL28-Halo
α-RPS3A
e
siControl
siSENP3-1
siSENP3-1 siSENP5-2
RPS3-Halo
40S
cytoplasmic intensity of new vs old ribosomes
siSENP3-1 + siSENP5-2
***
**

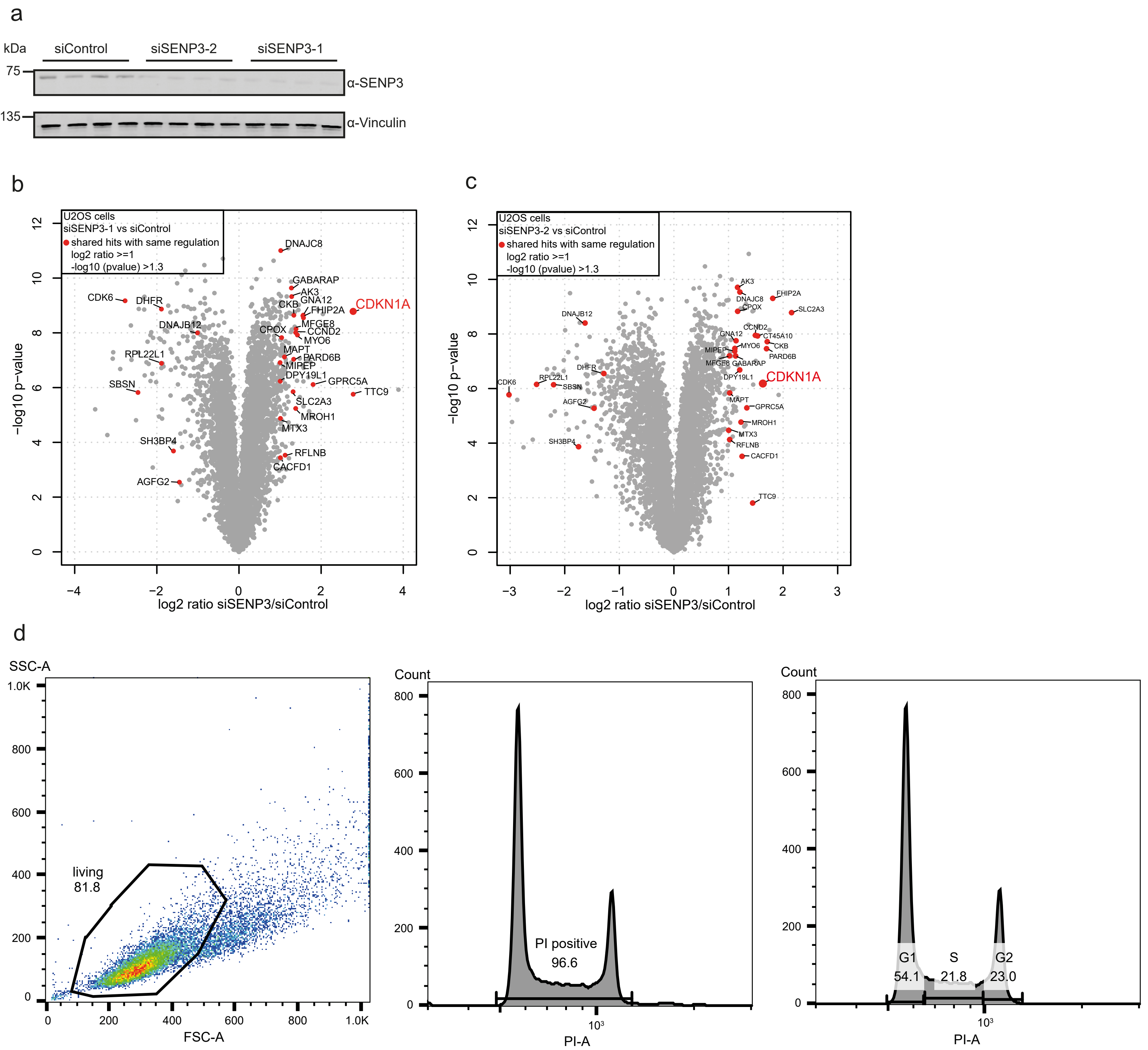
a
kDa
siControl
siSENP3-2
siSENP3-1
75
135
α-SENP3
α-Vinculin
b
U2OS cells
siSENP3-1 vs siControl
shared hits with same regulation
log2 ratio >=1
-log10 (pvalue) >1.3
−log10 p−value
log2 ratio siSENP3/siControl
DNAJC8
GABARAP
AK3
GNA12
CKB
FHIP2A
CDKN1A
CDK6
DHFR
DNAJB12
CPOX
MFGE8
CCND2
MYO6
MAPT
PARD6B
RPL22L1
MIPEP
DPY19L1
GPRC5A
SBSN
TTC9
SLC2A3
MROH1
MTX3
SH3BP4
RFLNB
CACFD1
AGFG2
c
U2OS cells
siSENP3-2 vs siControl
shared hits with same regulation
log2 ratio >=1
-log10 (pvalue) >1.3
−log10 p−value
log2 ratio siSENP3/siControl
d
SSC-A
FSC-A
living
81.8
Count
PI-A
PI positive
96.6
Count
PI-A
G1
54.1
S
21.8
G2
23.0

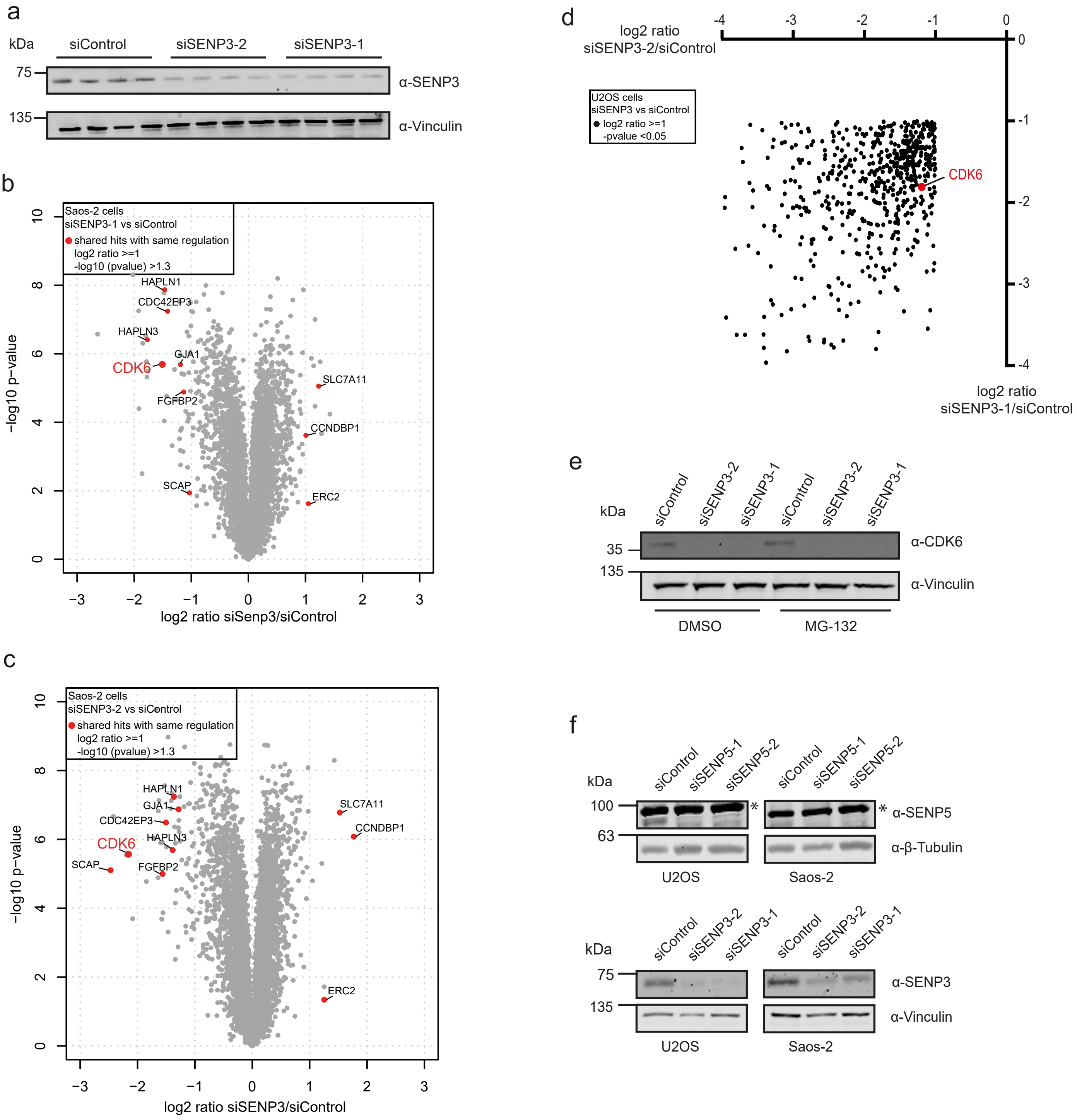
a
kDa
siControl
siSENP3-2
siSENP3-1
75
135
α-SENP3
α-Vinculin
b
Saos-2 cells
siSENP3-1 vs siControl
shared hits with same regulation
log2 ratio >=1
-log10 (pvalue) >1.3
HAPLN1
CDC42EP3
HAPLN3
GJA1
CDK6
FGFBP2
SLC7A11
CCNDBP1
SCAP
ERC2
−log10 p−value
log2 ratio siSenp3/siControl
c
Saos-2 cells
siSENP3-2 vs siControl
shared hits with same regulation
log2 ratio >=1
-log10 (pvalue) >1.3
HAPLN1
GJA1
CDC42EP3
CDK6
HAPLN3
SCAP
FGFBP2
SLC7A11
CCNDBP1
ERC2
−log10 p−value
log2 ratio siSENP3/siControl
d
log2 ratio
siSENP3-2/siControl
U2OS cells
siSENP3 vs siControl
log2 ratio >=1
-pvalue <0.05
CDK6
log2 ratio
siSENP3-1/siControl
e
kDa
siControl
siSENP3-2
siSENP3-1
siControl
siSENP3-2
siSENP3-1
35
135
α-CDK6
α-Vinculin
DMSO
MG-132
f
kDa
siControl
siSENP5-1
siSENP5-2
siControl
siSENP5-1
siSENP5-2
100
63
α-SENP5
α-β-Tubulin
U2OS
Saos-2
kDa
siControl
siSENP3-2
siSENP3-1
siControl
siSENP3-2
siSENP3-1
75
135
α-SENP3
α-Vinculin
U2OS
Saos-2

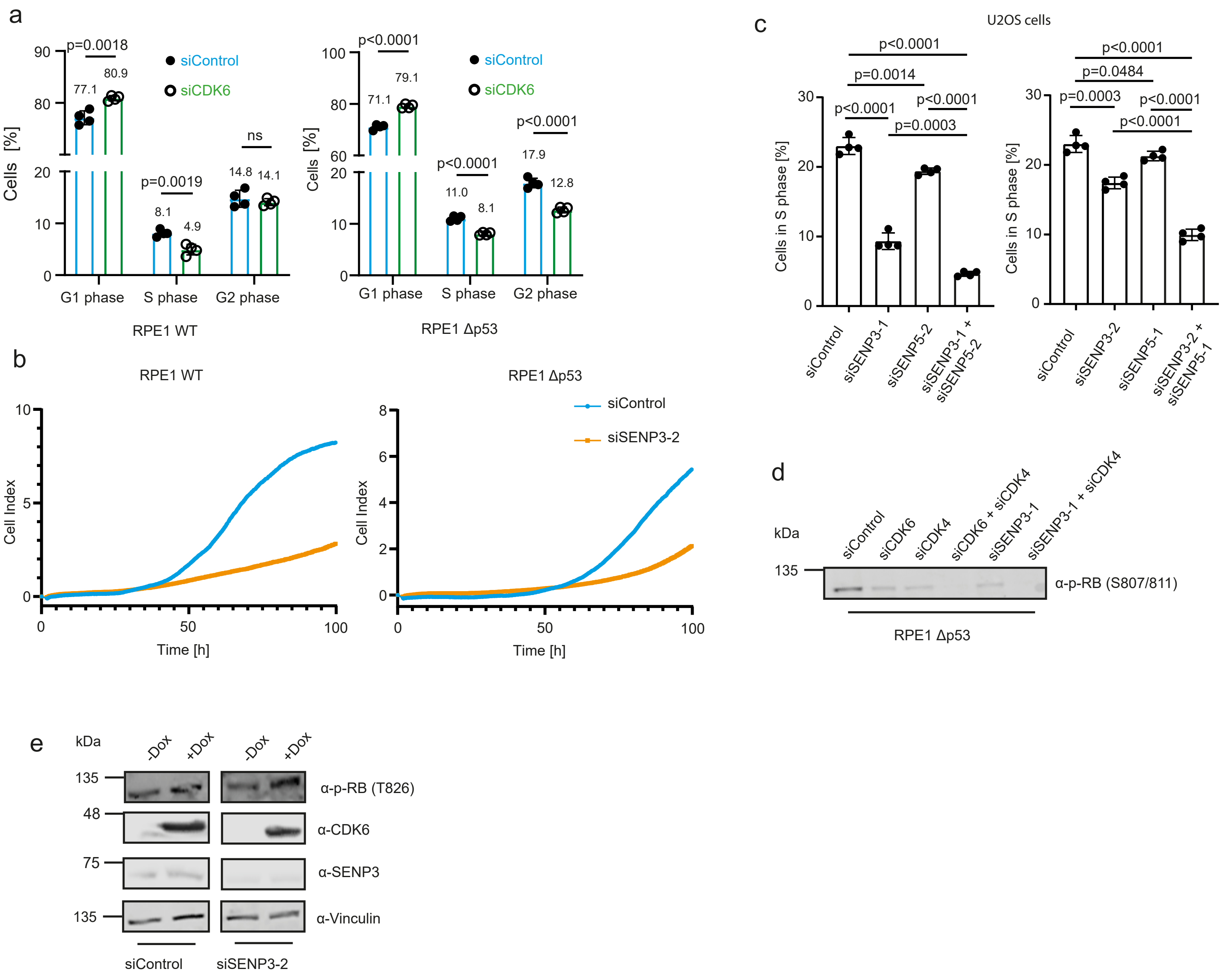
a
RPE1 WT
Cells [%]
siControl
siCDK6
p=0.0018
77.1
80.9
p=0.0019
8.1
4.9
ns
14.8
14.1
G1 phase
S phase
G2 phase
RPE1 Δp53
p<0.0001
71.1
79.1
p<0.0001
11.0
8.1
p<0.0001
17.9
12.8
b
RPE1 WT
RPE1 Δp53
Cell Index
Time [h]
siControl
siSENP3-2
c
U2OS cells
Cells in S phase [%]
p<0.0001
p=0.0014
p<0.0001
p<0.0001
p=0.0003
siControl
siSENP3-1
siSENP5-2
siSENP3-1 + siSENP5-2
p<0.0001
p=0.0484
p=0.0003
p<0.0001
p<0.0001
siControl
siSENP3-2
siSENP5-1
siSENP3-2 + siSENP5-1
d
kDa
135
siControl
siCDK6
siCDK4
siCDK6 + siCDK4
siSENP3-1
siSENP3-1 + siCDK4
α-p-RB (S807/811)
RPE1 Δp53
e
kDa
-Dox
+Dox
-Dox
+Dox
135
48
75
135
α-p-RB (T826)
α-CDK6
α-SENP3
α-Vinculin
siControl
siSENP3-2

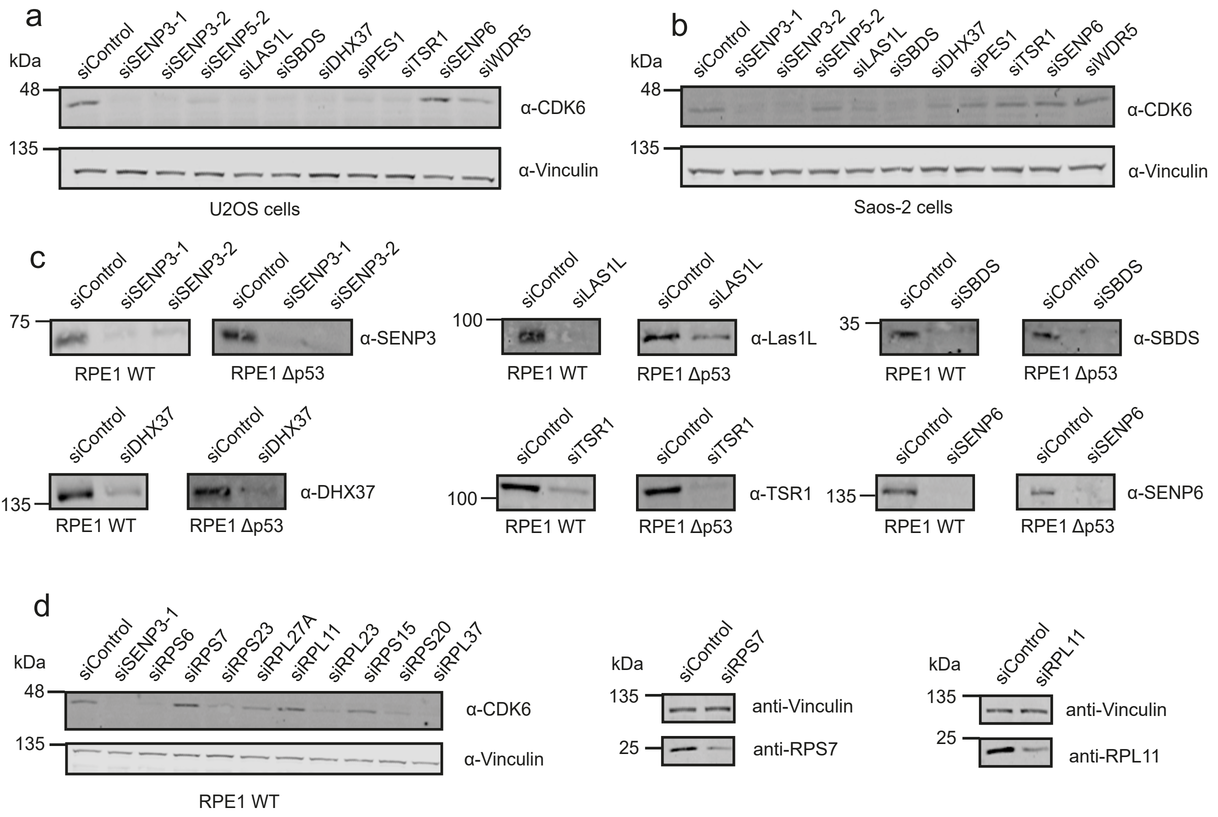
a
kDa
siControl
siSENP3-1
siSENP3-2
siSENP5-2
siLAS1L
siSBDS
siDHX37
siPES1
siTSR1
siSENP6
siWDR5
48
α-CDK6
135
α-Vinculin
U2OS cells
b
kDa
siControl
siSENP3-1
siSENP3-2
siSENP5-2
siLAS1L
siSBDS
siDHX37
siPES1
siTSR1
siSENP6
siWDR5
48
α-CDK6
135
α-Vinculin
Saos-2 cells
c
siControl
siSENP3-1
siSENP3-2
75
α-SENP3
RPE1 WT
RPE1 Δp53
siControl
siLAS1L
100
α-Las1L
siControl
siSBDS
35
α-SBDS
siControl
siDHX37
135
α-DHX37
siControl
siTSR1
100
α-TSR1
siControl
siSENP6
135
α-SENP6
d
kDa
siControl
siSENP3-1
siRPS6
siRPS7
siRPS23
siRPL27A
siRPL11
siRPL23
siRPS15
siRPS20
siRPL37
48
α-CDK6
135
α-Vinculin
RPE1 WT
siRPS7
anti-Vinculin
25
anti-RPS7
siRPL11
anti-RPL11
