# Supplementary material for: Characterization of nucleolar SUMO isopeptidases unveils a general p53-independent checkpoint of impaired ribosome biogenesis
Source: Nat Commun. 2023 Dec 8;14:8121. doi: 10.1038/s41467-023-43751-9 (PMC10709353; doi:10.1038/s41467-023-43751-9)
Supplement: Supplementary file 3 — Description of Additional Supplementary Files [file 41467_2023_43751_MOESM3_ESM.pdf]

Description of Additional Supplementary Files

File Name: Supplementary Data 1
Description: SENP3 and SENP5 interactomes

File Name: Supplementary Data 2
Description: Candidate SENP3-controlled SUMO targets

File Name: Supplementary Data 3
Description: Proteome for SENP3 targets

File Name: Supplementary Data 4
Description: Candidate SENP5-controlled SUMO targets

File Name: Supplementary Data 5
Description: Proteome for SENP5 targets

File Name: Supplementary Data 6
Description: UTP14A interactome

File Name: Supplementary Data 7
Description: Proteome for UTP14A interactome

File Name: Supplementary Data 8
Description: Proteome of siSENP3 in U2OS

File Name: Supplementary Data 9
Description: : RNA-Seq of siSENP3 in U2OS

File Name: Supplementary Data 10
Description: Proteome of siSENP3 in Saos-2

File Name: Supplementary Data 11
Description: RNA-Seq of siSENP3 in Saos-2

File Name: Supplementary Data 12
Description: List of used plasmids

File Name: Supplementary Data 13
Description: List of used siRNAs

File Name: Supplementary Data 14
Description: List of used antibodies
