# Supplementary material for: Characterization of nucleolar SUMO isopeptidases unveils a general p53-independent checkpoint of impaired ribosome biogenesis
Source: Nat Commun. 2023 Dec 8;14:8121. doi: 10.1038/s41467-023-43751-9 (PMC10709353; doi:10.1038/s41467-023-43751-9)
Supplement: Supplementary file 18 — Reporting Summary [file 41467_2023_43751_MOESM18_ESM.pdf]

nature portfolio

Corresponding author(s): Stefan Müller

Last updated by author(s): Oct 27, 2023

Reporting Summary

Nature Portfolio wishes to improve the reproducibility of the work that we publish. This form provides structure for consistency and transparency in reporting. For further information on Nature Portfolio policies, see our Editorial Policies and the Editorial Policy Checklist.

Statistics

For all statistical analyses, confirm that the following items are present in the figure legend, table legend, main text, or Methods section.

n/a	Confirmed	
☐	☒	The exact sample size (*n*) for each experimental group/condition, given as a discrete number and unit of measurement
☐	☒	A statement on whether measurements were taken from distinct samples or whether the same sample was measured repeatedly
☐	☒	The statistical test(s) used AND whether they are one- or two-sided<br>*Only common tests should be described solely by name; describe more complex techniques in the Methods section.*
☐	☒	A description of all covariates tested
☐	☒	A description of any assumptions or corrections, such as tests of normality and adjustment for multiple comparisons
☐	☒	A full description of the statistical parameters including central tendency (e.g. means) or other basic estimates (e.g. regression coefficient) AND variation (e.g. standard deviation) or associated estimates of uncertainty (e.g. confidence intervals)
☐	☒	For null hypothesis testing, the test statistic (e.g. *F*, *t*, *r*) with confidence intervals, effect sizes, degrees of freedom and *P* value noted<br>*Give P values as exact values whenever suitable.*
☐	☒	For Bayesian analysis, information on the choice of priors and Markov chain Monte Carlo settings
☐	☒	For hierarchical and complex designs, identification of the appropriate level for tests and full reporting of outcomes
☒	☐	Estimates of effect sizes (e.g. Cohen's *d*, Pearson's *r*), indicating how they were calculated

Our web collection on statistics for biologists contains articles on many of the points above.

Software and code

Policy information about availability of computer code

Data collection: No software was used for data collection

Data analysis:
ImageQuant software for quantification of Northern Blots (version 5.2)
Licor Image Studio Lite for quantification of Western Blots (version 5.2)
FlowJo for FACS data analysis (version 10)
For RNA-Seq data analysis following tools were used: Galaxy platform using the local server version of usegalaxy.org (RRID:SCR_006281). Adapter sequences were removed from FASTQ files using Trimmomatic (RRID:SCR_011848), DESeq2 (RRID:SCR_015687), GeneTrail3 (RRID:SCR_006250)
Fiji-BioVoxxel bundle in ImageJ (version 1.52i), CellProfiler (version 4.2.1) for IF data analysis
For MS data analysis following programms were used: MaxQuandt (version 2.4.2.0), Perseus (version 1.6.7.0), Proteom Discoverer (version 2.4)
R studio (version 4.1.2)
GraphPad PRISM (version 8.4.2)
ShinyGO (http://bioinformatics.sdstate.edu/go/ version 0.76.1)
STRING database (version 11.5)

For manuscripts utilizing custom algorithms or software that are central to the research but not yet described in published literature, software must be made available to editors and reviewers. We strongly encourage code deposition in a community repository (e.g. GitHub). See the Nature Portfolio guidelines for submitting code & software for further information.

Data

Policy information about availability of data

All manuscripts must include a data availability statement. This statement should provide the following information, where applicable:

- Accession codes, unique identifiers, or web links for publicly available datasets
- A description of any restrictions on data availability
- For clinical datasets or third party data, please ensure that the statement adheres to our policy

MS data were deposited on PRIDE.
Project Name: Identification of SENP3 and SENP5 target proteins by endogenous SUMO2/3 IP-MS; accession: PXD037793, PXD037796.
Project Name: Identification of UTP14A interactors by endogenous UTP14A IP-MS; accession: PXD043556.
Project Name: Whole cell proteome of U2OS cells or Saos-2 cells depleted for SENP3; Project accession: PXD037800.
RNA-Seq data have been stored at the European Nucleotide Archive and are accessible via accession ID: PRJEB57219

Research involving human participants, their data, or biological material

Policy information about studies with human participants or human data. See also policy information about sex, gender (identity/presentation), and sexual orientation and race, ethnicity and racism.

Reporting on sex and gender	N/A
Reporting on race, ethnicity, or other socially relevant groupings	N/A
Population characteristics	N/A
Recruitment	N/A
Ethics oversight	N/A

Note that full information on the approval of the study protocol must also be provided in the manuscript.

Field-specific reporting

Please select the one below that is the best fit for your research. If you are not sure, read the appropriate sections before making your selection.

☒ Life sciences ☐ Behavioural & social sciences ☐ Ecological, evolutionary & environmental sciences

For a reference copy of the document with all sections, see nature.com/documents/nr-reporting-summary-flat.pdf

Life sciences study design

All studies must disclose on these points even when the disclosure is negative.

Sample size	Sample sizes were chosen to ensure proper statsitical analysis as explained in the Material & Methods section and Figure legends.
Data exclusions	No data were excluded
Replication	All experiments (Western Blots, IPs, Northern Blots, Succrose gradients, immunofluorescence, FACS, qPCR, proliferation assays, MS, RNA-Seq) were perfomed with fully independent biological replicates to ensure correctness of the data. Large scale experiments as MS and RNA-Seq were performed with at least 3 replicates. Experiements like qPCR were additionally perfomed with technical replicates to minimize experimental errors.
Randomization	In all cell biological experiments cells were randomised to wells, and then wells randomized to treatments.
Blinding	For initial microscopic image analysis investigators were blinded to exclude any experimental bias-

Reporting for specific materials, systems and methods

We require information from authors about some types of materials, experimental systems and methods used in many studies. Here, indicate whether each material, system or method listed is relevant to your study. If you are not sure if a list item applies to your research, read the appropriate section before selecting a response.

Materials & experimental systems

n/a	Involved in the study	
☐	☒	Antibodies
☐	☒	Eukaryotic cell lines
☒	☐	Palaeontology and archaeology
☒	☐	Animals and other organisms
☒	☐	Clinical data
☒	☐	Dual use research of concern
☒	☐	Plants

Methods

n/a	Involved in the study	
☒	☐	ChIP-seq
☐	☒	Flow cytometry
☒	☐	MRI-based neuroimaging

Antibodies

Antibodies used

Anti-SUMO2/3, Hybridoma subclone A11 of 8A2 provided by F. Melchior (8A2 available fromDevelopmental studies hybridoma bank) clone 8A2, subclone A11

Anti-FLAG M2, Sigma Aldrich, F1804, RRID: AB_262044

Anti-UTP14A, Proteintech, 11474-1-AP, RRID:AB_2272800

Anti-β-Tubulin, Developmental Studies Hybridoma Bank, clone E7, RRID:AB_2315513

Anti-SENP3 (D20A10), Cell Signaling, #5591, RRID:AB_10694546

Anti-SENP5, Proteintech, 19529-1-AP, RRID:AB_10643377

Anti-LAS1L, Proteintech, 16010-1-AP, RRID:AB_2132810

Anti-PELP1, Proteintech, 67050-1-Ig, RRID:AB_2882363

Anti-RPL3, Proteintech, 11005-1-AP, RRID:AB_2181760

Anti-RPS3A, Proteintech, 14123-1-AP, RRID:AB_2253921

Anti-p53, Santa Cruz, sc-126, RRID:AB_628082

Anti-Vinculin, Sigma Aldrich, V9131, RRID: AB_477629

Anti-p21, Abcam, ab227443

Anti-CDK6, Cell Signaling, #13331, RRID:AB_2721897

Anti-SENP6, Sigma Aldrich, HPA024376, RRID: AB_1856678

Anti-CDK4, Santa Cruz, sc-56277, RRID:AB_1121419

Anti-RB (IF8), Santa Cruz, sc-102, RRID:AB_628209

Anti-p-RB T826, Abcam, ab-133446, RRID:AB_2722666

Anti-p-RB S807/811, Cell Signaling, #9308, RRID:AB_331472

Anti-SUMO2/3, MBL, M114-3, clone 1E7, RRID:AB_592769

Anti-UTP18, ThermoFisher Scientific, PA5-90468, RRID:AB_2806090

Anti-BMS1, Santa Cruz, sc-271040, RRID:AB_10611468

Anti-RPL28, Abcam, ab138125

Anti-RPS3, Santa Cruz, sc-376008

Anti-Anti-HaloTag® Monoclonal Antibody, Promega, G9211

Anti-RPS9, Aviva Systems Biology Corp, ARP61782_P050

Anti-SBDS, Sigma Aldrich, HPA028891, RRID: AB_10601500

Anti-DHX37, Bethyl, A300-856A-T, RRID:AB_2292875

Anti-TSR1, Proteintech, 16887-1-AP, RRID:AB_2222589

Anti-RPS7, Abcam, ab 57637, RRID:AB_945322

Anti-RPL11, Abcam, ab79352, RRID:AB_2042832

Anti-RGS-His, Quiagen, 34650, RRID:AB_2687898

IRDye® 800CW Goat anti-Mouse IgG Secondary Antibody, Li-Cor, 926-32210, RRID: AB_621842

IRDye® 800CW Goat anti-Rabbit IgG Secondary Antibody, Li-Cor, 926-32211, RRID: AB_621843

Validation

Anti-SUMO2/3, Hybridoma subclone A11 of 8A2: application IP, validation PMID: 24651501

Anti-FLAG M2: application western blot and IF, validation PMID: 31399583 PMID: 31900387 PMID: 32034124

Anti-UTP14A: application western blot, IF and IP, validation PMID: 33391409 PMID: 37086406 PMID: 27798105

Anti-β-Tubulin: application western blot, validation PMID: 32200800 PMID: 31833223 PMID: 31328806

Anti-SENP3: application western blot, validation PMID: 29277914 PMID: 31141694 & in this manuscript by siRNA knockdown

Anti-SENP5: application western blot, validation PMID: 29277914 & in this manuscript by siRNA knockdown

Anti-LAS1L: application western blot, validation by siRNA knockdown in this manuscript

Anti-PELP1: application western blot, validation PMID: 37290329, PMID: 21326211 and manufacturer's website

Anti-RPL3: application western blot, validation PMID: 34725147 PMID: 34081545 PMID: 35674491

Anti-RPS3A: application western blot, validation PMID: 29518358 PMID: 30612743 PMID: 31825839

Anti-p53: application western blot, validation PMID: 30879903 PMID: 30057196 PMID: 30415951 & in this manuscript by p53 pathway activation

Anti-Vinculin: application western blot, validation PMID: 29576454 PMID: 29053959 PMID: 27984728

Anti-p21: application western blot, validation PMID: 35696596 PMID: 33000191 PMID: 32897806 & in this manuscript by p53 pathway activation

Anti-CDK6: application western blot, validation PMID: 30840889 PMID: 31056462 PMID: 30300583 & in this manuscript by siRNA knockdown

Anti-SENP6: application western blot, validation PMID: 31597105 & in this manuscript by siRNA knockdown

Anti-CDK4: application western blot, validation PMID: 29225033 & in this manuscript by siRNA knockdown

Anti-RB (IF8): application western blot, validation PMID: 35859155 PMID: 34584098

Anti-p-RB T826: application western blot, validation PMID: 30300583 PMID: 30454645

Anti-p-RB S807/811: application western blot, validation PMID: 30197300 PMID: 30380421 PMID: 30454645

Anti-SUMO2/3: application western blot, validation PMID: 30581133 PMID: 31141694 & in this manuscript by WB following IP

Anti-UTP18: application western blot, validation on manufacturer's website

Anti-BMS1: application western blot, validation PMID: 10659855 PMID: 12477932 PMID: 15635413

Anti-RPL28: application western blot, validation PMID: 32612236 PMID: 30712990 PMID: 29230017

Anti-RPS3: application western blot, validation PMID: 16737853 PMID: 17560175 PMID: 18610840

Anti-Anti-HaloTag® Monoclonal Antibody: application western blot, validation PMID: 37591251, PMID: 37708126, PMID: 36791199

Anti-RPS9: application western blot, validation on manufacturer's website

Anti-SBDS: application western blot, validation PMID: 31597105 & in this manuscript by siRNA knockdown

Anti-DHX37: application western blot, validation PMID: 31442407 & by antibody provider & in this manuscript by siRNA knockdown

Anti-TSR1: application western blot, validation PMID: 29503206 & in this manuscript by siRNA knockdown

Anti-RPS7: application western blot, validation in this study by siRNA knockdown

Anti-RPL11: application western blot, validation in this manuscript by siRNA knockdown

Anti-RGS-His, Quiagen, 34650, RRID:AB_2687898, validation PMID: 34022140, PMID: 32521226, PMID: 28803726

IRDye® 800CW Goat anti-Mouse IgG Secondary Antibody, Li-Cor, 926-32210, validation on manufacturer's website

IRDye® 800CW Goat anti-Rabbit IgG Secondary Antibody, Li-Cor, 926-32211, validation on manufacturer's website

Eukaryotic cell lines

Policy information about cell lines and Sex and Gender in Research

Cell line source(s)	U2OS (human, female, ATCC HTB-96)<br>SAOS-2 (human, female, ATCC HTB-85)<br>HeLa (human, female, ATCC CCL-2)<br>HEK293T (human,female, ATCC CRL-3216)<br>RPE1 (human, female, ATCC CRL-4000)<br>BxPC3 (human, female, ATCC CRL-1687)<br>Ramos (human, male, ATCC CRL-1596)
Authentication	U2OS, SAOS-2, HeLa, HEK293T, RPE1, BxPC3 and Ramos cells were authenticated by STR profiling
Mycoplasma contamination	All cell lines were tested negative for mycoplasma contamination
Commonly misidentified lines<br>(See ICLAC register)	No commonly misidentified cell line was used in this study

Plants

Seed stocks	*Report on the source of all seed stocks or other plant material used. If applicable, state the seed stock centre and catalogue number. If plant specimens were collected from the field, describe the collection location, date and sampling procedures.*
Novel plant genotypes	*Describe the methods by which all novel plant genotypes were produced. This includes those generated by transgenic approaches, gene editing, chemical/radiation-based mutagenesis and hybridization. For transgenic lines, describe the transformation method, the number of independent lines analyzed and the generation upon which experiments were performed. For gene-edited lines, describe the editor used, the endogenous sequence targeted for editing, the targeting guide RNA sequence (if applicable) and how the editor was applied.*
Authentication	*Describe any authentication procedures for each seed stock used or novel genotype generated. Describe any experiments used to assess the effect of a mutation and, where applicable, how potential secondary effects (e.g. second site T-DNA insertions, mosiacism, off-target gene editing) were examined.*

Flow Cytometry

Plots

Confirm that:

- [x] The axis labels state the marker and fluorochrome used (e.g. CD4-FITC).
- [x] The axis scales are clearly visible. Include numbers along axes only for bottom left plot of group (a 'group' is an analysis of identical markers).
- [x] All plots are contour plots with outliers or pseudocolor plots.
- [x] A numerical value for number of cells or percentage (with statistics) is provided.

Methodology

Sample preparation	RPE1 WT, RPE1 Δp53, BxPC3, Ramos or U2OS cells were grown in 6-well plates under standard conditions and transfected

	with siRNA for 72 h. Cells were trypsinized and washed with PBS. Afterwards cells were fixed using an ice-cold 70% [v/v] EtOH solution and incubated for 1 h on ice. Following an additional washing step with PBS, cells were incubated with FACS-stain solution (0.5% Triton X-100, 20 µg/ml PI, 20 µg/ml RNase A in PBS) for 1 h to stain the DNA and digest remaining RNA. DNA content was measured by flow cytometry.
Instrument	BD FACSCantoTM II, BD Biosciences
Software	Data were collected using the BD FACSDiva™ Software. Afterwards, data were analysed by FlowJo (FlowJo, LLC).
Cell population abundance	At least 10000 living cells were gated and considered for further analysis.
Gating strategy	Cells were plotted with the FSC-A against the SSC-A channel. The living population was gated and then plotted for the PI-A signal as a histogram. Only PI positive cells were gated. G1, S and G2 phases were determined based on the PI signal. The same gates were transferred to all samples in the same experiment.

☒ Tick this box to confirm that a figure exemplifying the gating strategy is provided in the Supplementary Information.
